# Induced Magnetic Field-Based Indoor Positioning System for Underwater Environments

**DOI:** 10.3390/s21062218

**Published:** 2021-03-22

**Authors:** Sizhen Bian, Peter Hevesi, Leif Christensen, Paul Lukowicz

**Affiliations:** 1 Embedded Intelligence, German Research Center for Artificial Intelligence (DFKI), 67663 Kaiserslautern, Germany; peter.hevesi@dfki.de (P.H.); paul.lukowicz@dfki.de (P.L.); 2 Embedded Intelligence, Technische Universitaet Kaiserslautern, 67663 Kaiserslautern, Germany; 3 Robotics Innovation Center, German Research Center for Artificial Intelligence (DFKI), 28359 Bremen, Germany; leif.christensen@dfki.de

**Keywords:** magnetic field, magnetic induction, underwater positioning, AUV positioning and navigation, indoor positioning

## Abstract

Autonomous underwater vehicles (AUV) are seen as an emerging technology for maritime exploration but are still restricted by the availability of short range, accurate positioning methods necessary, e.g., when docking remote assets. Typical techniques used for high-accuracy positioning in indoor use case scenarios, such as systems using ultra-wide band radio signals (UWB), cannot be applied for underwater positioning because of the quick absorption of the positioning medium caused by the water. Acoustic and optic solutions for underwater positioning also face known problems, such as the multi-path effects, high propagation delay (acoustics), and environmental dependency. This paper presents an oscillating magnetic field-based indoor and underwater positioning system. Unlike those radio wave-based positioning modalities, the magnetic approach generates a bubble-formed magnetic field that will not be deformed by the environmental variation because of the very similar permeability of water and air. The proposed system achieves an underwater positioning mean accuracy of 13.3 cm in 2D and 19.0 cm in 3D with the multi-lateration positioning method and concludes the potential of the magnetic field-based positioning technique for underwater applications. A similar accuracy was also achieved for various indoor environments that were used to test the influence of cluttered environment and of cross environment. The low cost and power consumption system is scalable for extensive coverage area and could plug-and-play without pre-calibration.

## 1. Introduction and Related Work

Over the past decades, the importance of the world’s oceans for the economy has grown steadily. Although the sea has long played a crucial role in trade, transportation, and fishing, humans are utilizing increasingly more areas of the ocean for extended applications, including advancing to ever greater depths. While the oil and gas industry was a main driver in the past decades for maritime know-how (and continues to invest in emerging technologies), other sectors have a higher potential for sustainable growth. The European Commission, for example, names aquaculture, coastal tourism, marine biotechnology, ocean energy, and seabed mining in this context [1]. Although Germany plays only a minor role in the worldwide maritime economy, the German Association of Shipbuilding and Maritime Technology (VSM) estimates an annual revenue of around 13.4 billion dollars for the maritime technologies sector in Germany alone [2]. All of these sectors require significant advances of the state-of-the-art in maritime technology, both to enhance efficiency as well as to become more sustainable in the future. Robotic systems like remotely operated vehicles (ROVs) and especially autonomous underwater vehicles (AUVs) are seen as a game changer for exploration and exploitation of the marine environment [3], AUVs are still only adopted in niche applications and have yet to be utilized in broader fields [4].

One of the main challenges for AUVs and sub-sea operations in general is the restricted availability of established localization methods in comparison to terrestrial applications. While Global Navigation Satellite Systems (GNSS; e.g., GPS) are the standard reference technology for surface localization there, these systems are not applicable in the underwater domain, due to the strong attenuation of higher frequency electromagnetic signals [5].

Underwater positioning technique is traditionally addressed by the medium of acoustics [6,7] because of the long-range propagation of the acoustic wave in the water. The underwater object’s position is derived using acoustic range measurements (time of flight) involving known anchor or surface nodes. However, the speed of sound in water is a complex function that varies with temperature, salinity, and depth [8], and there is the multi-path effects when the acoustic wave propagates underwater. Plenty of potential underwater positioning approaches has been developed in recent years as summarised by Wu et al. [9] and Tariq et al. [10]. For example, Morgado et al. [11] validated the inertial navigation system for underwater vehicles and got an enhanced performance of error estimation regarding the accumulated drift caused by the inertial components. Shuang et al. [12] proposed a vision-based framework by addressing the detection and pose estimation problems for short-range underwater docking. Jia et al. [13] utilized an omnidirectional camera to locate and navigate underwater vehicles by computing the differences between the expected and the recognized position at each time and forward the difference information to the attitude adjusting center. Vision-based underwater positioning supplies cm-level accuracy, whereas an in-advance calibration is needed because of its sensitivity to the environmental variation. The optic signal is another medium utilized for underwater positioning profiting from its fast speed. The large bandwidth also enables the underwater communication. Farhad et al. [14] proposed an underwater optical positioning system. An anchor node transmits visual signals. The sensor nodes receive the optical signals from multiple anchors and locate themselves using a simple linear least square solution. The practical tests of the proposed optical approach show the localization performance with root mean square error of 0.8 m and 1.6 m using Time-of-Flight (ToF) and Received Signal Strength (RSS), respectively [15]. In practical applications, regarding the advantages of each technique in the coverage area, positioning accuracy, deployment cost, centralized/decentralized demand, and environmental dependency, the fusion of different underwater positioning and navigation techniques is utilized. An example is Simultaneous localization and mapping (SLAM), a positioning and navigation technology that uses multi-sensory information (camera, light, and LiDAR). Numerous studies in the field of underwater robots have recently focused on SLAM [16,17,18], which might be the most potential approach to achieve completely autonomous underwater robot positioning and navigation. Combining different sensory data derived from disparate underwater sensing modalities is a robust way to accomplish different underwater application scenarios (for an overview see [19]), but due to the inhomogeneity of the water column, localization remains a major challenge for sub-sea operations [20]. Especially close range navigation tasks like sub-sea asset inspection and maintenance or docking maneuvers require sophisticated setups [21], tactical grade sensors [22] or even pre-installed infrastructure like long baseline (LBL) acoustic transponders.

In this paper, we present a novel underwater positioning and navigation method utilizing the induced low-frequency magnetic field. As a first trial step of addressing the challenge for close range navigation, this work mainly verify the potential feasibility from the induced magnetic field. Compared with the above-listed approach, the induced magnetic field is a silent medium without multi-path effects as there are no RF-like propagating waves. The motivation of this work comes from our experience of induced magnetic field based proximity and indoor positioning work and the very similar permeability of water and air. Our previous low-frequency magnetic field based-work for indoor location [23,24] and social distance monitoring [25,26] has demonstrated the robustness of resonating magnetic field to the everyday environment. While the magnetic permeability of surrounding high-conductive material may distort the local ambient magnetic field, especially in robotic applications (for an overview, see in [27]), these distortions may often be compensated for by a priori-calibration or by utilizing machine learning techniques as described in [28]. Under special circumstances, the local field distortions may also be used for navigation purposes in underwater docking and homing [29].

Previous underwater magnetic-based research from literature mainly focused on underwater communication [30,31,32], sub-sea cable tracking [33] with geomagnetic field anomalies. Underwater positioning based on the induced magnetic field was rarely described in the literature. Jonas et al. [34] explored the usage of tri-axial magnetometers and a friendly vessel with known magnetic dipole to silently localize the sensors and conclude a sensor positioning accuracy of 26.1% with simulated data. Akyildiz et al. [32] also mentioned and roughly described magnetic induction-based underwater positioning in their communication-focused paper; however, detailed experiment and performance data were not described. In comparison, our work provides a detailed description of the design, implementation, and evaluation of the resonant magnetic field-based system for indoor and underwater positioning.

In this paper, motivated by the potential of the technology within marine applications described above, we optimized our previous work on oscillating magnetic field positioning for indoor environments for better accuracy and evaluated its performance in the underwater environment. Overall, we present the following contributions in this paper:

1. We optimized the triaxial magnetic antenna (coils) regarding our previous work [23,24], significantly decreased the crosstalk of the magnetic field generated in each axis. We also redesigned the signal processing unit at the magnetic field strength sensing side and decreased the noise, thus improved the positioning accuracy.
2. We evaluated the magnetic-based positioning system in a swimming pool and compared the performance to full-equipped office room with chairs, tables, desktop computers, a social place with empty space, and a household. Evaluations show that the system can provide the positioning accuracy of 15.3 cm indoors and 13.3 cm underwater in 2D and 18.0 cm indoors and 19.0 cm underwater in 3D.
3. The system is low cost, low power consumption, scalable for extensive coverage area, and could plug-and-play without pre-calibration.

This paper is structured as follows: Section 1 described the motivation for our work and related work from literature. The physical background and measurement principle were stated in Section 2. This section also described the hardware implementation, especially the coils at both transmitter and receiver sides, and the core part of signal processing. Indoor and underwater evaluations of the system’s positioning performance were then stated in Section 3. Section 4 concluded our work and illustrated the future work.

## 2. Approach

### 2.1. Physical Principle

In our previous magnetic field-based social distance monitoring work [26], we made a detailed introduction about the hardware system’s physical principle (as well as the system structure, circuit layout, and signal processing chain), here we briefly summarized the critical point for a constructive understanding of our hardware system. According to Maxwell’s electromagnetic theory [35], the degradation of the magnetic field strength is inversely proportional to the cube of distance, as described by Equation (1), where $\vec{B}$ is magnetic field strength with the unit of Tesla, $\mu_{0}(4\pi \;*\;10^{-7}$) is the permeability in the vacuum, *n* is the number of turns of the coil, *I* is the current with the unit of ampere flowing into the coil, *a* is the radius of the coil with the unit of meter, and $\vec{z}$ is the distance in meters from to be measured point to the center of the coil. A sinusoidal-varying magnetic field will be activated when a sinusoidal current is flowing in the coil. The strength of the magnetic was determined by current frequency, amplitude, the turns of the coil, and the permeability of the medium. According to Faraday’s law of induction, when we put another coil inside the bubble-like varying magnetic field, an induced voltage will occur in the new coil. By measuring the induced voltage amplitude, the distance information between the two coils can be derived, which constructs our induced magnetic field-based indoor and underwater positioning system’s essential working background.

(1) $${\vec{B}}_{\vec{z}}=\frac{\mu_{0}na^{2}I}{2{\sqrt{\left(a^{2}+{\vec{z}}^{2}\right)}}^{3}}$$

### 2.2. Transmitter and Receiver Coils

**Figure 1:**
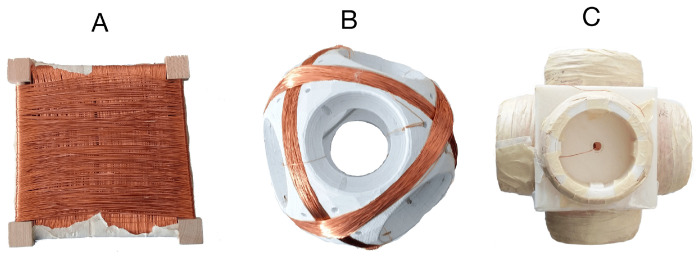
Evaluated manually twined coils. (**A**) The initial cubic design (17 cm in length, 20 mH) with high crosstalk due to large overlapping coil areas, (**B**) an intermediate round version (15 cm in diameter, 18.6 mH) with lower cross talk due to reduced overlapped area, and (**C**) the current version (18 × 18 × 18 cm in size and 33 mH in inductance of each axis).

**Figure 2:**
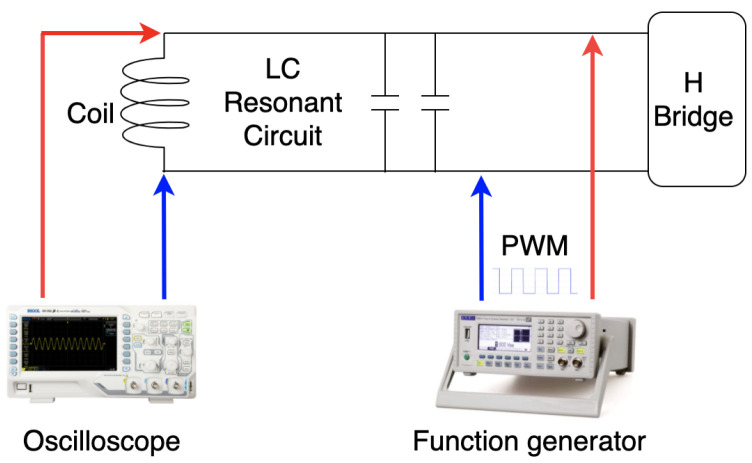
Calibration of LC resonant circuit, where the resonant inductor refers to coils at both transmitter and receiver sides.

The coils are the most critical component in magnetic field generating (transmitter antenna) and strength sensing (receiver antenna), thus we have put a lot of effort into the coils’ design. Figure 1 shows the development history of the transmitter coils, which work as the magnetic field generator in a resonant circuit. All transmitter coils are manually twined with painted copper wires (0.8 mm in diameter) on the 3D-printed frame structures. The coil’s inductance value is calculated in a resonant circuit with the help of the function generator and the oscilloscope, as Figure 2 depicts. By triggering the resonant circuit with the Pulse-width modulation (PWM) signal (with the sweeping test frequencies), the Pk-to-Pk voltage value on the coil will reach its highest when the sweeping frequencies come to the resonant one, and the inductance of the coil can be derived by the values of resonant capacitance and frequency. The H-Bridge is used to trigger the resonant circuit to generate the magnetic field during the practical applications and is disconnected while doing the coil calibration. Once we got all transmitter coils with the same inductance value, we calibrated the resonant capacitors until an LC resonant circuit with a resonant frequency of 20 kHz was achieved. The same method was also applied to the receiver coils. In the current hardware system, the LC resonant circuit at both transmitter and receiver sides is tuned with the resonant frequency of 20 kHz. As future work, the frequency could be enlarged to strengthen and broaden the magnetic field. To be noticed, the 20 kHz frequency is the resonant frequency of the LC circuit, which also means that the activated magnetic field occurs in space with the frequency of 20 kHz.

**Figure 3:**
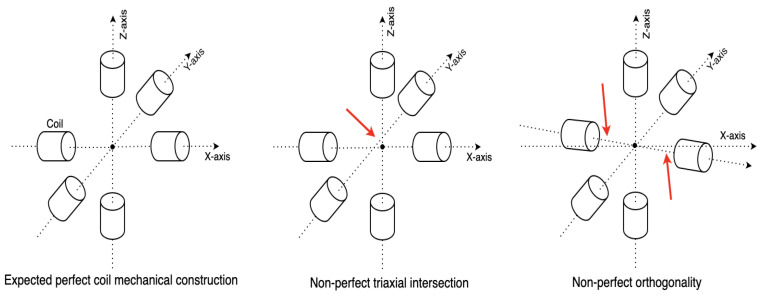
Cross-talk still exist because of non-perfect triaxial intersection and non-perfect orthogonality.

**Figure 4:**
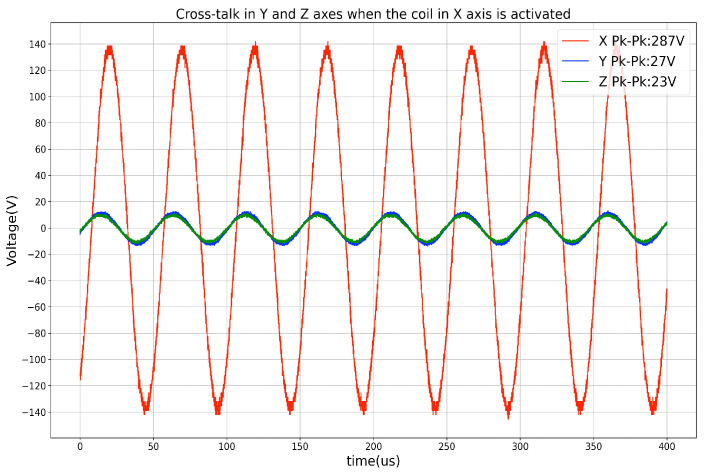
Cross-talk (represented by Peak-To-Peak voltage) on Y and Z axis while X axis being activated.

The initial magnetic antenna design has high cross-talk voltages between the coils when the magnetic field from a single axis is activated. The cross-talk was measured by the oscilloscope when triggering the transmitter coil in one axis with the function generator and observing the voltage on the other two axes’ coils. To ensure that the magnetic field is entirely generated by the transmitter’s single-axis coil, the cross-talk needs to be as low as possible. As the depiction in Figure 1A, because of the overlapping and short distance between the wires of the different axes, both inductive and capacitive cross-talks exist, which result in the superposing of magnetic fields and therefore affects the overall field layout. The inductive cross-talk is an electromagnetic induction phenomenon for all systems that utilize alternating current. The level of inductive coupling between two coils hugely depends on their shape, relative orientation, and distance. To reduce the effects of inductive cross-talk, each axis of the final new transmitter architecture (Figure 1C) consists of two sub-coils with the copper wire strictly parallel to each other. The coils of the other two axes are placed orthogonally to the existing coil. The capacitive cross-talk occurs when energy is coupled from one circuit to another through an electric field. In fact, any wires/cables with electrons flowing through will generate a static electric field around, which results in electrons redistribution in other nearby wires/cables. The capacitive coupling will be significantly reduced by increasing the distance between the wires. The new transmitter coil architecture is designed by eliminating the overlapped area and enlarging the coils’ relative distance in the three axes. The three axes of the transmitter are structured with the best effort to be orthogonal. However, as the coils are manually twined and taped to the 3D-Printer printed frame structure, the cross-talk still exists, as Figure 3 depicts. There are two reasons for this: the non-perfect mechanical orthogonality and the non-perfect triaxial intersection. Table 1 shows the average cross-talk decreasing from more than 60% to less than 10%. The crosstalk was measured by activating the coil at each transmitter axis and observing the peak-to-peak voltage on the coils from the other axes, as Figure 4 depicts (with data exported from the oscilloscope). The inductance of each axis in the final transmitter design is around 33 mH. By tuning the serially connected pF-level capacitors, a coupled LC circuit with expected oscillating frequency is formed, acting as the magnetic field generation unit.

**Figure 5:**
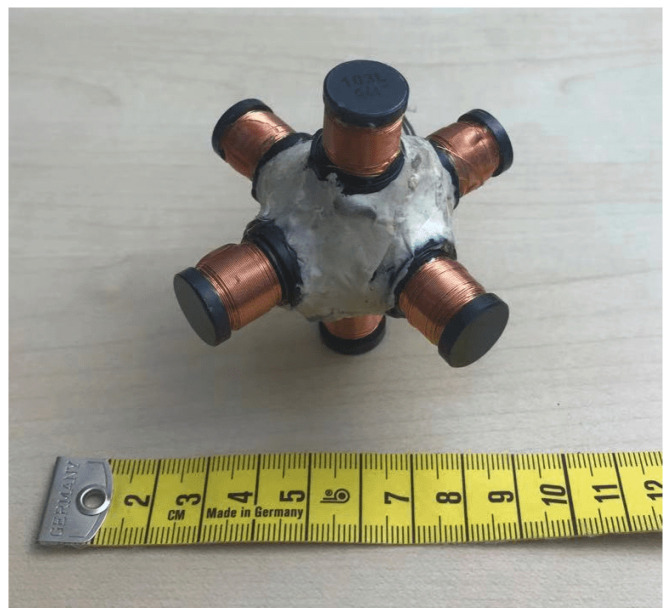
Receiver coil (8.5 × 8.5 × 8.5 cm and 6.6 mH in inductance of each axis).

The same designing method was also applied to the receiver coil, as Figure 5 depicts. The receiver coils were also designed in a cube form and composed of coils in three axes with two serially connected inductors in each axis. The inductance of each axis of the receiver coils is 6.6 mH. A similar coupled LC oscillating circuit is then tuned to sense the magnetic field strength nearby.

### 2.3. System Architecture

**Figure 6:**
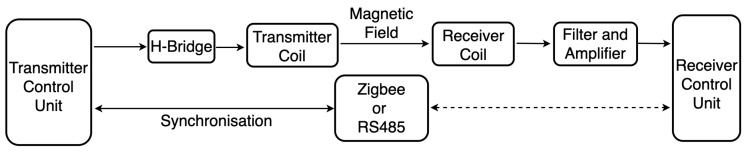
System architecture composed of transmitter system, receiver system, and the synchronization unit.

**Figure 7:**
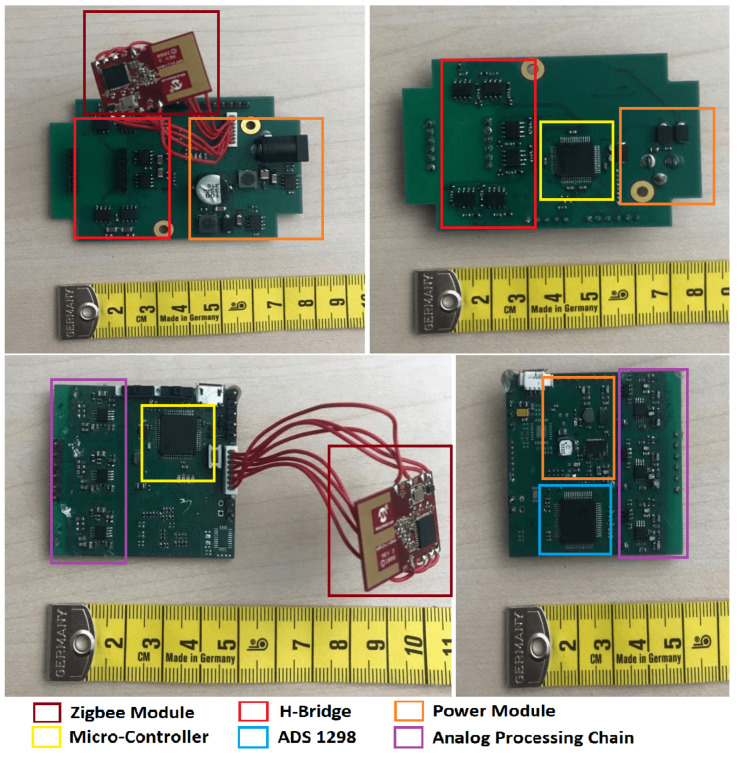
Transmitter and receiver PCBs with labeled modules.

Figure 6 illustrates the prototype’s hardware architecture. The transmitter control unit drives the H-bridge to activate the magnetic field on the resonant coil by a coded PWM signal. The receiver coil with the same resonant frequency is then triggered by the surrounding magnetic field and forwards the induced resonant voltage to the signal processing unit, which is mainly composed of a 4-order Butter–Worth filter and a logarithmic amplifier. The processed signal is then sampled by a 24-bit high-resolution analog-to-digital chip for further data evaluation. The hardware cost is less than fifty dollars altogether, which is extremely cheap compared with other underwater positioning equipment on the market. Another cost is mainly from the work of coil manufacture as all coils were made manually. The synchronization of multiple transmitters was addressed by an extra unit where Zigbee (wirelessly, for the indoor environment) or RS485 protocol (wired, mainly for the underwater environment) was used to book the coils’ working status for each transmitter. More details will be described in Section 2.4. Figure 7 depicts the transmitter and receiver PCB with a Zigbee module on them. While the system is in operation, the receiver system consumes 120 mA current with a 3.7 V power supply. The transmitter system consumes 170 mA current with a 10 V power supply.

**Figure 8:**
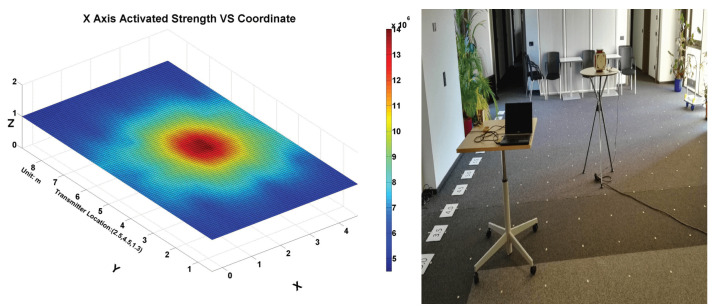
(**Left**): Rectified magnetic field strength planar distribution after interpolation. (**Right**): A social place with 0.5 m grids where the data was collected.

Figure 8 shows the distribution of magnetic field strength in the planar space. As described in the physical principle section, the strength signal is not a RF-like propagating wave, but a bubble-like field. Thus, the limitation in wave-based positioning approach, like refraction, reflection, and multi-path effects, does not exist in our magnetic field-based positioning approach. The data were collected while only the x-axis of a transmitter coil was activated. It is obvious from the figure that the magnetic field strength falls quickly with the rising distance. To be noticed, the strength depicted in the figures of this work is not the practical magnetic field strength with the unit of Tesla. Instead, we use the raw value from the analog to digital converter as the indirect indication of the strength for further evaluation, which is proved to be feasible as there a mathematical relationship between the practical strength and sampled rectified strength. However, the relationship is hard to derive with a back–forward inference (especially when there is a logarithmic amplifier in our hardware signal processing chain) and is not necessary regarding our final purpose of positioning.

### 2.4. Transmitter Synchronization and Identification

The rectified magnetic strength data sampled by the receiver system needed to be synchronized to the activated transmitter axis. We use two approaches: Zigbee as a wireless approach and RS485 as a wired approach to address this issue.

#### 2.4.1. Zigbee for Wireless Approach

**Figure 9:**
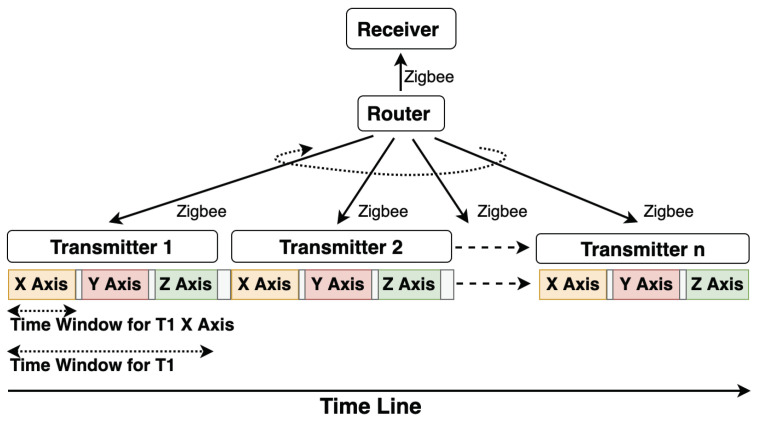
Synchronization with Zigbee module where a router Zigbee broadcasts the ID of the next transmitter, which is going to activate three magnetic field sequentially.

**Figure 10:**
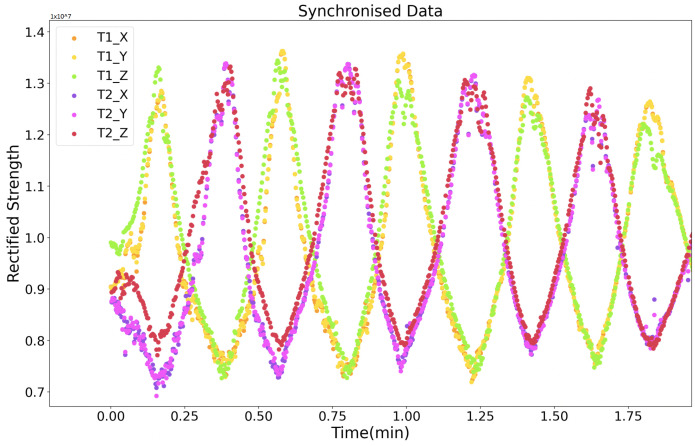
Rectified strength of two transmitters with Zigbee module as the synchronization medium.

The Zigbee module broadcasts the identification of the next being activated transmitter. The magnetic field from each transmitter axis has the same time length, so the receiver “knows” whose magnetic field data is being sampled. As Figure 9 depicts, a router Zigbee broadcasts the ID of the transmitters with a certain interval, and the ID is listened to by all transmitters and the receiver. Once a transmitter gets its ID, it activates its axes to generate the magnetic fields sequentially. The receiver starts sampling for a certain time (slightly less than the Zigbee’s broadcast period) as long as an ID is received, then stops sampling and starts listening for the next ID. Within each axis’s time window, the receiver samples at least three times to get an averaged strength value. Finally, the receiver’s raw data include both the magnetic field strength information and the corresponding transmitter ID, as Figure 10 presents for an instance of two transmitters with Zigbee communication mechanism. To be noticed, the router Zigbee can be a separate module or any Zigbee module already mounted on any transmitters or the receiver.

#### 2.4.2. RS485 for Wired Approach

**Figure 11:**
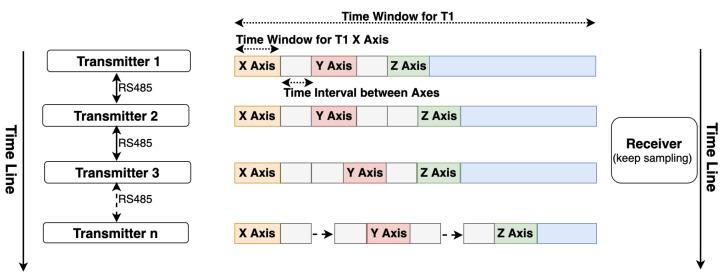
Synchronization with RS485 protocol where each transmitter performs the magnetic field activation once a “start” order was received through RS485 protocol. The receiver recognize the transmitter ID by observing the time gap between the detected strength peaks.

**Figure 12:**
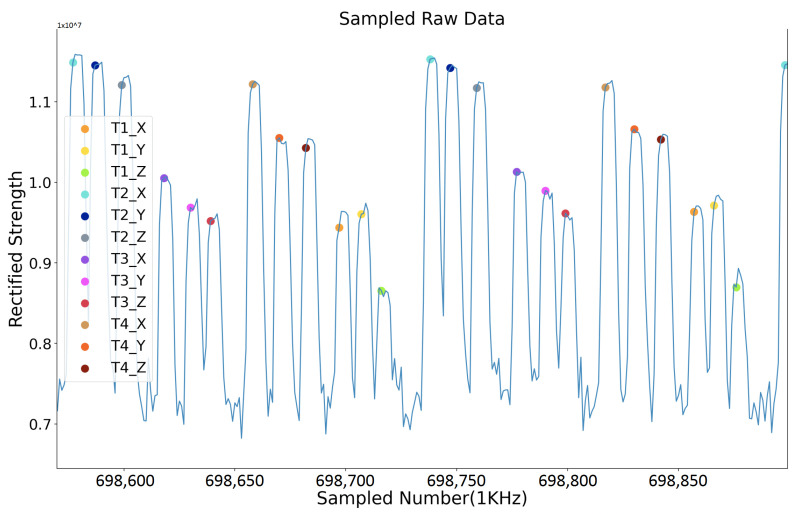
Example of synchronization result with RS485 protocol in a four-transmitter configuration.

**Figure 13:**
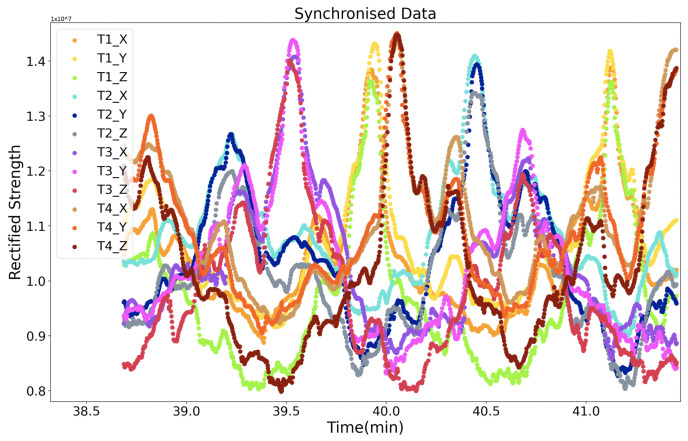
Rectified strength with only magnetic-related data (four transmitters with RS485 module).

A second synchronization mechanism is based on the RS485 protocol, where each transmitter starts to sequentially activate its axes and generate three magnetic fields sequentially once a “start” order from the RS485 cable is received. After its time window, the transmitter gives a “start” order to its cascaded transmitter. This mechanism’s critical point is that the three sequentially activated magnetic fields’ intervals are different for each transmitter. This mechanism is used when doing the underwater positioning where Zigbee does not work. Unlike the Zigbee approach, where the current activated transmitter’s ID is directly supplied to the receiver, this RS485-based approach needs to obtain the ID information from the sampled strength data. As Figure 11 depicts, the receiver recognizes the current transmitter by the interval time between each sampled magnetic field strength data period. This approach requires a high sampling rate at the receiver side. Figure 12 gives a shot of the preprocessed data sampled at the receiver side after the synchronization, where four transmitters were deployed in the environment. The first step is to calculate the amplitude of raw data sensed by the receiver (as the receiver coil also has three axes). Then, a peak detection method is applied to pick up the strength data. The final step is to recognize the peaks’ belonging by observing the peaks’ time interval. Figure 13 shows the trend of the twelve magnetic fields strength from the four transmitters after synchronization and identification process on the raw data. Our next step is to abstract the distance information from the magnetic field strength data.

#### 2.4.3. Scalability of the System

To cover a larger area for positioning, the system needs to be scalable. The first solution is that the single transmitter’s magnetic field range should be as large as possible. Our current hardware configuration can supply a magnetic field with a detectable range of 3.5 m in radius. This range can be significantly enlarged by improving the oscillating frequency, enlarging the coil area, or supplying higher current, as inferred by Equation (1). Some primary studies are described in Section 4 as future work. The second approach is to deploy more transmitters. Although every transmitter needs a certain time window to be activated and hold the magnetic field for a certain time so that the receiver can sample the strength data, more transmitters do not decrease the update rate. Once the distance of two transmitters is beyond the detectable magnetic field radius, the two transmitters could be activated simultaneously. This simultaneous activation applies to both the Zigbee-based and RS485-based synchronization mechanisms.

### 2.5. Preliminary Disturbance Test

**Figure 14:**
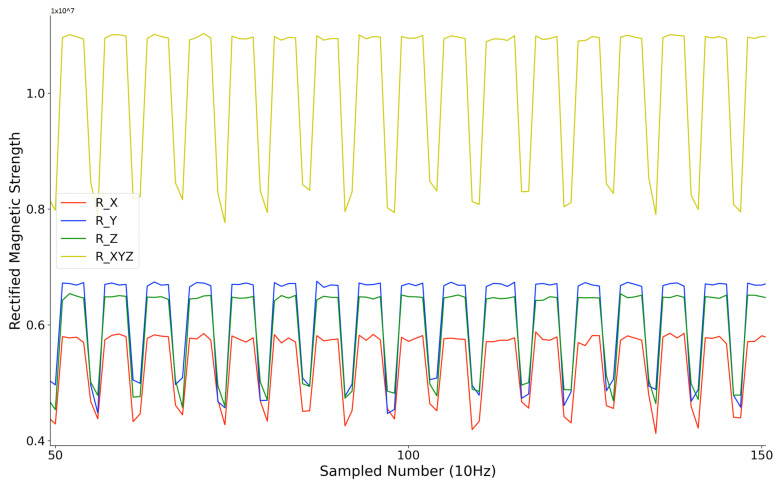
Received rectified strength from the three axes of receiver with a 400 ms activation-window and 200 ms non-activation-window of the transmitter.

Compared to other beacon-type systems, our system’s key advantage is that it has no RF-like wave propagation, which means no non-negligible reflection, no refraction, etc. exists. Due to the very close relative permeability of water and air (0.99999976 vs. 1.00000037), the submersion of the system in deeper water should not change the field signal’s properties. The signal changes only if within the range of non-negligible magnetic field (which is limited to a few meters because of the $r^{3}$ decrease and the size of the coils) there is an object in which the magnetic field can induce a current (typically eddy current) losing energy/inducing an opposing field. To experimentally test the influence from the maritime environment on the distribution of the magnetic field strength, we performed a simple test where we put different medium between the transmitter and receiver (near the receiver with less than 1 cm distance, on the path in between with more than 70 cm distance to both transmitter and receiver, near the transmitter with less than 1 cm distance), and observe the rectified strength. The transmitter was configured to a single axis magnetic field generator, with the activation window of 400 ms and a non-activation window of 200 ms. As Figure 14 shows, the sensed raw rectified magnetic field strength was plotted. Their combination was calculated by performing the square root calculations of the sum of square of each axis.

**Figure 15:**
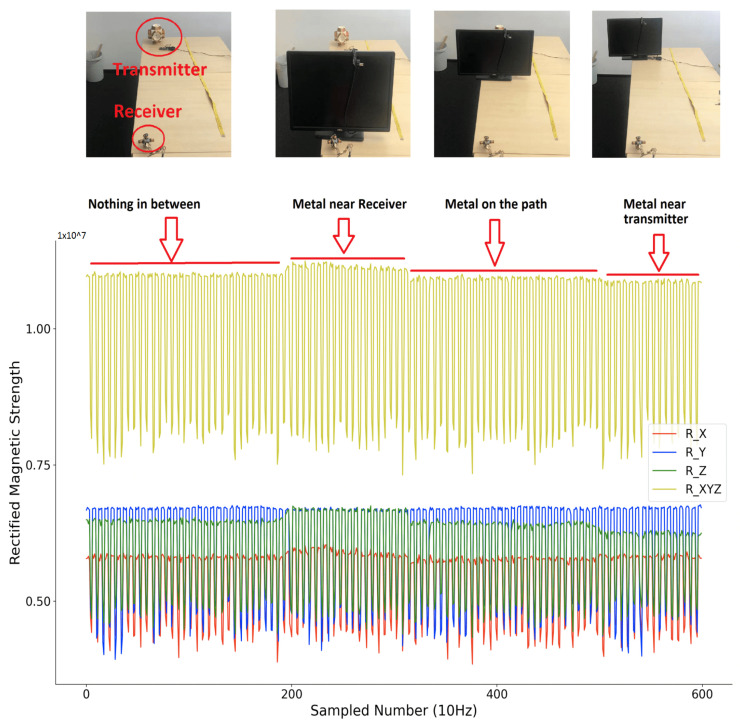
Sensed magnetic field rectified strength (metallic object in between).

**Figure 16:**
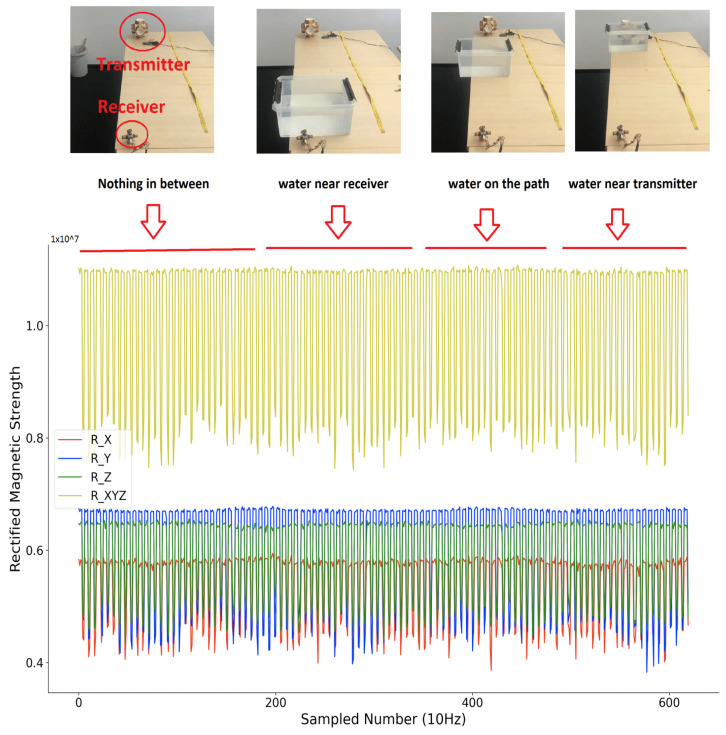
Sensed magnetic field rectified strength (water in between).

**Figure 17:**
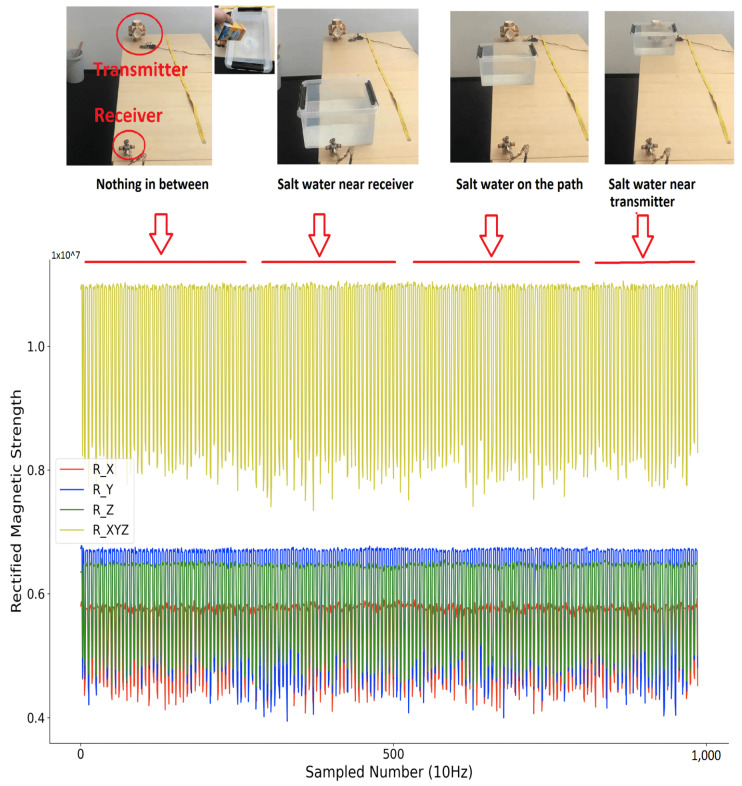
Sensed magnetic field rectified strength (salt water in between).

Figure 15, Figure 16 and Figure 17 depict the sensed rectified strength when putting metallic object, water, and saltwater (with an average salinity of 3.5%, as a simulation of the oceanic water) on the path between transmitter and receiver with different distance, respectively. From the sensed raw data of the rectified magnetic field strength, it is obvious that only a metallic object that is very close to the receiver or transmitter will deform the sensed rectified strength. The water and saltwater do not significantly impact the field distribution. Note that if the receiver is mounted on a metal arm of the underwater vehicle, simple calibration for the disturbance will address the metallic disturbance, as the envelope of the strength signals in Figure 15 shows.

## 3. Evaluation

**Figure 18:**
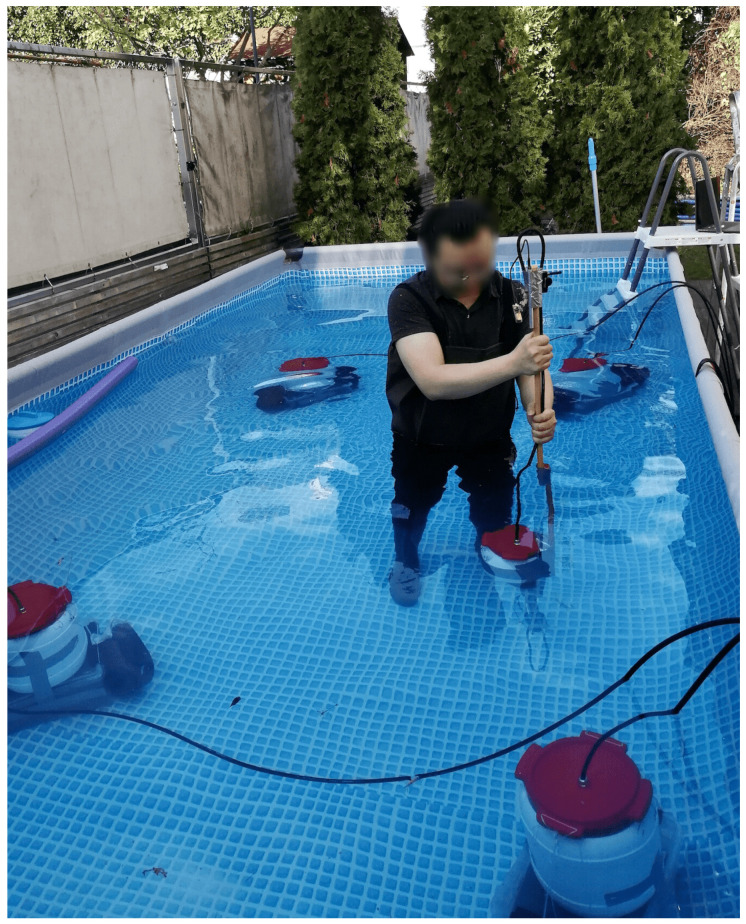
Underwater evaluation experiments in a swimming pool (549 × 274 × 122 cm), where four RS-485 synchronized transmitters were placed at the bottom of the pool.

**Figure 19:**
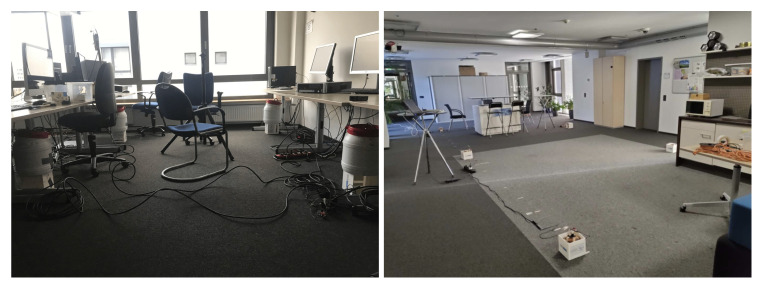
Indoor experiment performed as a baseline to compare with the underwater accuracy. Working office (**left**, 4 × 4 m), social place (**right**, 5 × 4 m).

To evaluate our underwater magnetic field-based system’s positioning performance, we did experiments in a swimming pool with both the transmitters and the receivers submerged (Figure 18). In addition, we conducted an in-depth evaluation at two standard indoor environments, a large office (student working area) and the social meeting place in our lab (Figure 19). The aim of the indoor experiments was threefold. First, in the indoor environment it was easier to have variety of obstacles (tables, chairs, and desktop computers) and evaluate the influence of such obstacles on the signal. Second, in the office environment we were able to use a baseline “gold standard” ultrasound system for ground truth allowing a more dynamic and precise data collection. Finally, they allowed us to investigate the impact of using different environments for calibrating the systems and for deploying them. Given the difficulty of underwater data collection, this is a relevant aspect for the submarine domain.

In the office room and underwater experiments, the transmitters were connected by cables and synchronized by RS485 protocol. In the social place experiment, Zigbee was used to synchronize all transmitters and the receiver. Four transmitters were used during the experiments (for a successful positioning, at least three transmitters are needed to utilize the trilateration approach. Although the transmitter has three axes, they supply similar field distribution in space, as Figure 10 and Figure 13 depict). The orientation of all transmitters was kept the same. The layout of transmitters was randomly chosen and could be in any shape. The only limitation is that each to be positioned point needs to be in the range of the detectable radius of at least three transmitters. The ground-truth was supplied by the products from MarvelMind Robotics [36], which provides a set of ultrasound hardware system being able to give indoor positioning and navigation data with a precise of 2 cm. The ultrasound system could supply stable 3D positioning information in the indoor environment. However, in the outdoor underwater environment, the z-axis data lost the ground-truth information. The reason behind this could be the complexity of the environment in the vertical dimension. As a result, we performed the underwater experiment by moving the receiver in a planar way and with six different receiver heights. The height data were recorded as the ground-truth of the z-axis. Am ultrasound router collected the ultrasound data to the computer, and the magnetic field strength data were transferred from the receiver to the computer through the Bluetooth module of ESP32 [37].

**Figure 20:**
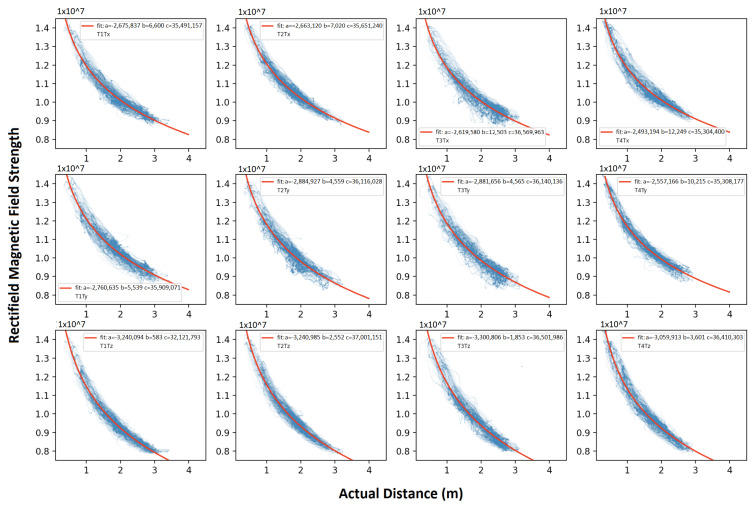
Fitted relationship of each magnetic field rectified strength and actual distance (office experiment).

**Figure A1:**
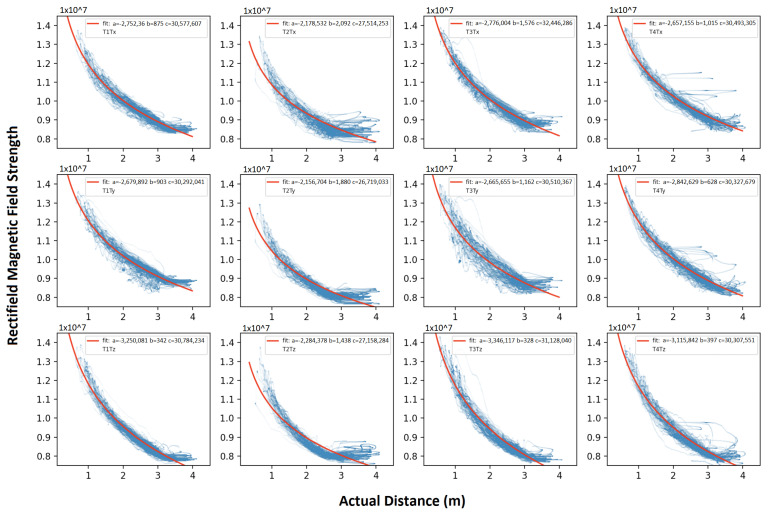
Fitted relationship of each magnetic field rectified strength and actual distance (social place experiment).

**Figure A2:**
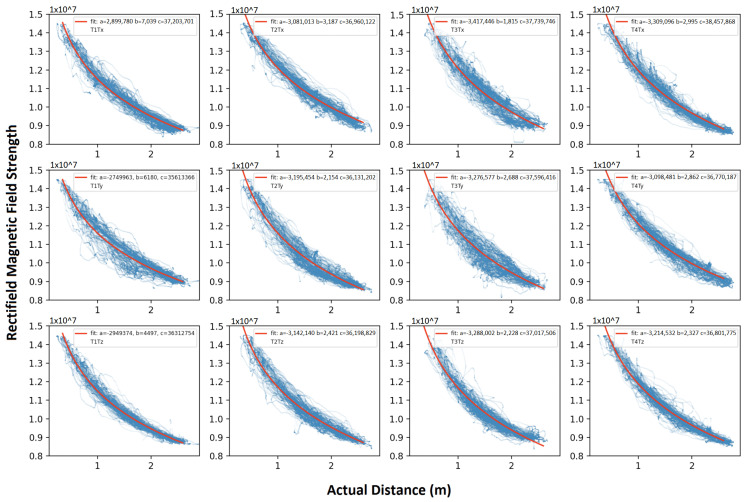
Fitted relationship of each magnetic field rectified strength and actual distance (underwater experiment).

As data from both the ultra-sound router and receiver Bluetooth were transferred to the computer, we assigned the timestamp to the data to synchronize the ground-truth to each magnetic field’s strength information. In the four transmitters configuration, we assigned each transmitter a 55 ms time window, which means a 4.55 Hz update rate of strength for each transmitter. Within the time window, for the RS485 synchronization approach, each axis was activated for 6 ms. The interval between the axis’s activation window was 3 ms or 6 ms, depending on the transmitter ID. For the Zigbee synchronization approach, each axis was activated for 10 ms successively with 5 ms gap time and then listened to the next “start” order. The ground-truth data were supplied with an update rate of 8 Hz. We interpolated both ultrasound and magnetic fields’ time-series data to an array with 100 Hz sample rate. Then, we were able to get the relationship of rectified magnetic field strength generated by each axis of the transmitter and the practical distance, as Figure 20 depicts. The practical distance was received by calculating the Euclidean distance between the receiver coordinates and the corresponding transmitter coordinates. Considering the actual strength–distance relation (Equation (1)) and the signal processing unit on the receiver board (Butterworth filter and logarithmic amplifier), we used a curve-fitting method to describe the relation of rectified strength and actual distance with

(2) $$B_{rec}=a*10^{-b*D}+c$$

where $B_{rec}$ is the rectified strength; *D* is the actual distance; and a,b,c are three parameters to be determined by the fitting process. Figure 20 gives the fitting result of the magnetic field from each axis while deploying the transmitters in the office. As all coils were manually manufactured, the parameters like inductance, turns, area, could not be guaranteed, all fields were fitted with slightly different parameters, although they all show very similar trend. The fitting result of social place and underwater experiment are displayed in Figure A1 and Figure A2.

Once we get the fitting equation, we then use the multi-lateration method to estimate the receiver’s position based on the sensed rectified strength. Multi-lateration algorithm computes the receiver’s most probable coordinates when the coordinates of a finite number of transmitters and the distances of the receiver to each transmitter are given. Our experiments used four cubic transmitters, thus twelve distances and transmitter coordinates were used to locate the receiver. Even if the distances computed for each transmitter coordinate do not match, the algorithm will find the coordinate that minimizes the error function and returns the most optimal solution possible. We use the “minimize” library [38] from the Python Scipy package to realize this multi-lateration method and get the estimated position. A Savitzky–Golay-Filter [39] is finally used to smooth the estimated data with a window size of 20 s.

**Figure 21:**
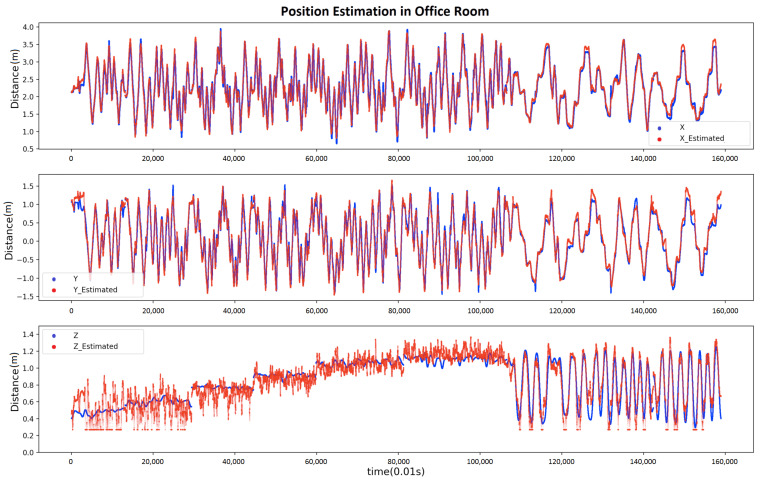
Comparison of the true coordinate with the estimated coordinate (office experiment).

**Figure A3:**
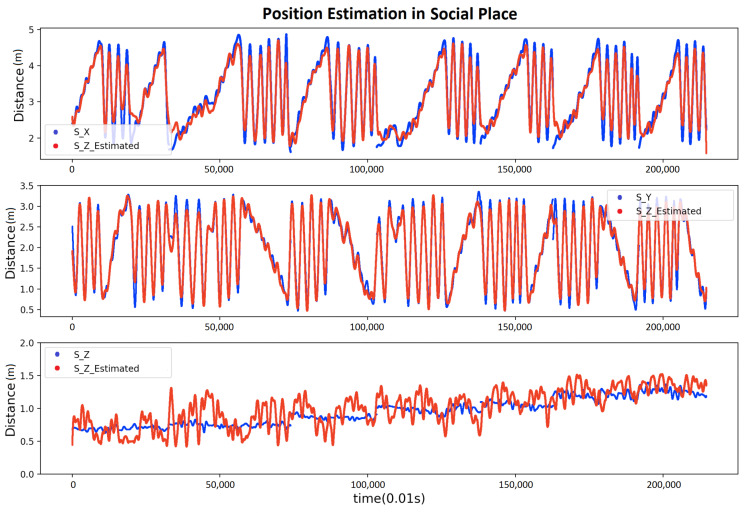
Comparison of the true coordinate with the estimated coordinate (social place experiment).

**Figure A4:**
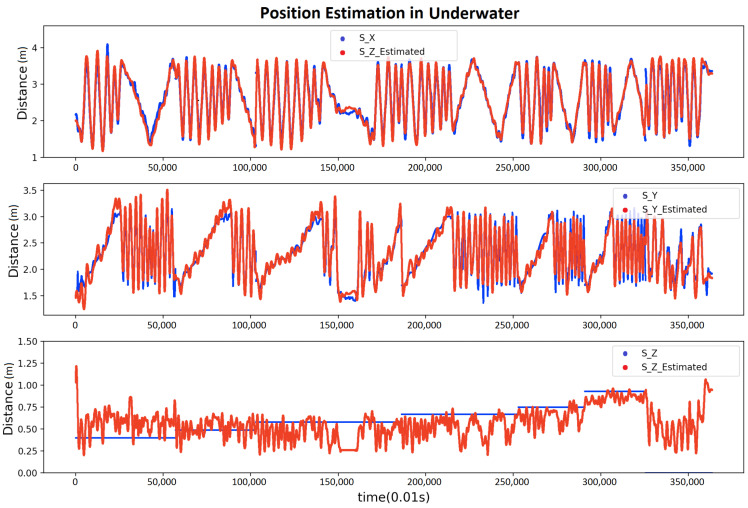
Comparison of the true coordinate with the estimated coordinate (underwater experiment).

Figure 21 shows the estimation result compared with the actual position in the office room. The ultrasound system supplies the actual position. During the experiment, the volunteer held the receiver system in hand and moved in a planar way in a random direction with different heights for around 1100 s. Then, the volunteer moved the receiver in any direction in 3D space, including varying the vertical height continuously. Figure A3 and Figure A4 show the estimation result compared with the actual position in the social place and underwater, respectively. There is one point to be clarified, during the underwater experiment, the ultrasound system could not provide sufficient Z-axis ground truth; thus, we only recorded the height of the receiver as the ground truth while moving the receiver in a planar way from start to around 3250 s. After the planar data collection, the volunteer moved the receiver randomly in any direction in 3D space continuously (as the end part of Figure A4 depicts). As the height information couldn’t be recorded during this period, we did not consider data from this period while evaluating the estimation performance.

Table 2 lists the mean absolute error (MAE) and standard deviation (Std) of the positioning estimation in the three environments with the parameters abstracted from their own data for the fitting Equation (2). The result is competitive among similar magnetic field-based positioning work, like in [40], where the positioning errors will accumulate to meter-level while fused with IMU; in [41], where the average error is 0.3 m in an office environment; and in [42], where the mean localization error is 0.8 m in 3D and 0.4 m in 2D.

**Figure 22:**
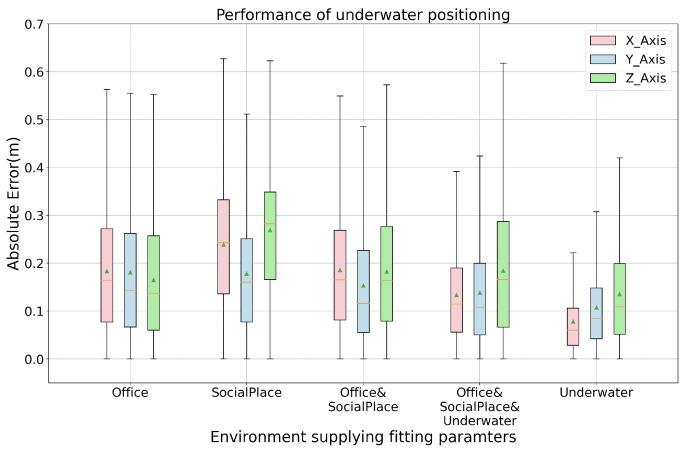
Underwater positioning result with the fitting parameters from different environment.

The positioning result in the underwater environment shows competitive positioning accuracy, around 0.133 m in 2D (x-y-plane) and 0.190 m in 3D (x-y-z-space). To test the generality of the system’s positioning performance in a different environment, we did cross-estimation to observe the performance, namely, abstracting the fitting parameters from data collected in other environments and then calculate the estimated positioning with the data from the to-be-evaluated environment. Table 3 lists the cross-estimation result and the combine-estimation result where all data was combined to abstract the fitting parameters. Compared with Table 2, the cross-evaluated positioning accuracy drops when using parameters abstracted from other environments. However, the results also show that a set of robust and general fitting parameters could be obtained with more data. As Figure 22 depicts, the underwater positioning with the office and social place data as a base could still get the mean absolute error less than 20 cm in each space axis. When combining all collected data to abstract the fitting parameters, the evaluated positioning result is competitive to the best evaluation result when abstracting the fitting parameters from the locally collected data only. This means that our system does not need to collect data for fitting parameter abstraction whenever the system is deployed to a new environment. In other words, the system can provide a plug-and-play positioning service when the positioning accuracy demand does not exceed 20 cm.

## 4. Conclusions and Future Work

The results that we have presented confirm that oscillating magnetic field systems are a promising short range (a few meters) positioning technology for submarine applications that require accuracy in the range of 10 to 20 cm. This can be used to support maneuvering and manipulating objects in low-visibility conditions where camera based tracking is limited to very short range. In such conditions they can allow the system to accurately approach a target to within visibility range. The experiments in indoor environments further demonstrated robustness with respect to clutter in the environment which is relevant for many applications.

A key limitation of this study is that verification has been performed in a shallow swimming pool only, not in real oceanic environment. As has been explained at length and illustrated in experiments (Section 2.5), because our signal does not rely on wave propagation but on the extension and retraction of a magnetic field, there is little reason to believe that the performance will be significantly different in a deep salt water basin. An additional indication of the likely performance in deep salt water is the fact that the physical principle is analogous to inductive near field communication for which has been widely studied for ocean environment (see, e.g., in [43]. Nonetheless, we acknowledge that a definite proof of performance in deep salt water will required an experiment in a oceanic environment. This is part of our future work. We would also like to point out that the current positioning is more through parameter fitting rather than model-based. Which is not necessarily a significant problem, but clearly a deeper model grounding will be in future work investigated.

**Figure 23:**
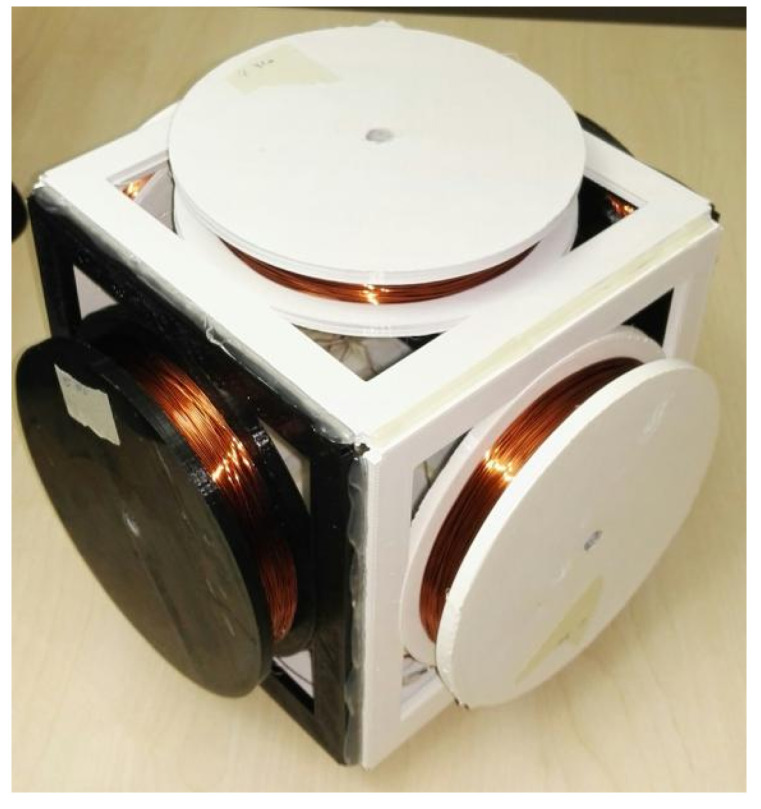
Our latest design of transmitter magnetic field antenna (19 × 19 × 19 cm, 28 mH).

**Figure 24:**
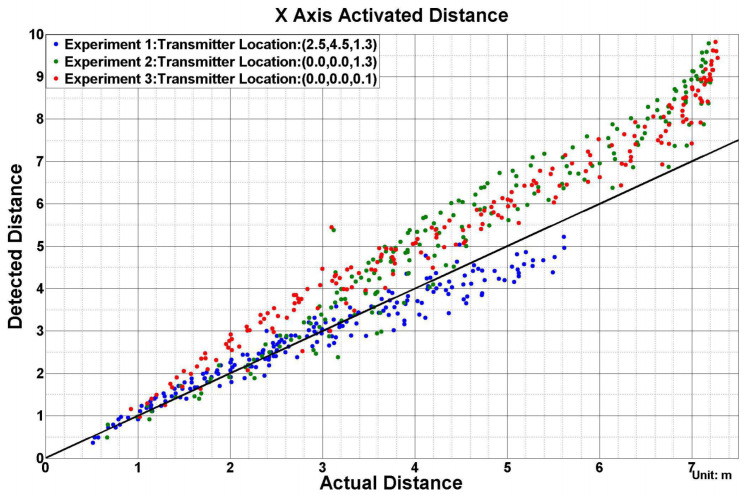
Coverage radius of the latest transmitter design.

In future work, we will also aim to increase the spatial coverage of single transmitter at the hardware side. This mainly depends on the transmitter coils’ design, like the Q-factor, and circular coil radius. The driver side parameters also play roles, like the oscillating frequency and the applied voltage on the LC-circuit. Some preliminary work has already demonstrated the improvement space for the coverage area. As Figure 23 depicts, when we enlarged the radius of the coil and increased the driving voltage from 8 V to 22 V, the detectable magnetic range was improved to 7 m in radius, as Figure 24 depicts. We will also explore dynamic combination of magnetic field and very short range vision based localization for very precise guidance in low visibility conditions and perform experiments under real world submarine scenarios.

## Figures and Tables

**Table 1:** Peak-to-peak voltage in each coils (unit: V).

Transmitter Coil	Activated Axis	Voltage on X	Voltage on Y	Voltage on Z
Figure 1A	X	232	155 (63%)	137 (58%)
	Y	158 (68%)	245	179 (67%)
	Z	132 (57%)	167 (68%)	236
Figure 1C	X	287	21 (8%)	30 (11%)
	Y	27 (9%)	270	24 (9%)
	Z	23 (8%)	27 (10%)	272

**Table 2:** Positioning result (MAE(Std)) in the three environments (unit: m).

Environment	X	Y	Z
Underwater	0.078 (0.073)	0.108 (0.091)	0.136 (0.106)
Office	0.099 (0.071)	0.117 (0.085)	0.095 (0.078)
Social Place	0.164 (0.149)	0.116 (0.093)	0.153 (0.101)

**Table 3:** Cross/Combine-Positioning result (MAE(Std)) in the three environments (unit: m).

Env-A ${}^{1}$	Env-B ${}^{2}$	X	Y	Z
Underwater	Office	0.184 (0.128)	0.181 (0.146)	0.165 (0.123)
Underwater	SocialPlace	0.239 (0.131)	0.179 (0.125)	0.269 (0.134)
Underwater	SocialPlace & Office	0.186 (0.128)	0.154 (0.129)	0.183 (0.126)
Underwater	Underwater & SocialPlace & Office	0.133 (0.101)	0.139 (0.116)	0.184 (0.135)
Office	SocialPlace	0.280 (0.142)	0.117 (0.087)	0.245 (0.149)
Office	Underwater	0.121 (0.082)	0.152 (0.115)	0.135 (0.087)
Office	SocialPlace & Underwater	0.097 (0.075)	0.124 (0.096)	0.111 (0.078)
Office	SocialPlace & Underwater & Office	0.097 (0.074)	0.117 (0.090)	0.101 (0.073)
SocialPlace	Office	0.258 (0.166)	0.220 (0.143)	0.233 (0.152)
SocialPlace	Underwater	0.315 (0.187)	0.195 (0.134)	0.170 (0.122)
SocialPlace	Underwater & Office	0.285 (0.165)	0.188 (0.129)	0.162 (0.108)
SocialPlace	SocialPlace & Underwater & Office	0.227 (0.148)	0.154 (0.110)	0.169 (0.110)

^1^ Environment of the test data. ^2^ Environment where the fitting parameters are abstracted.

**Figure A5:**
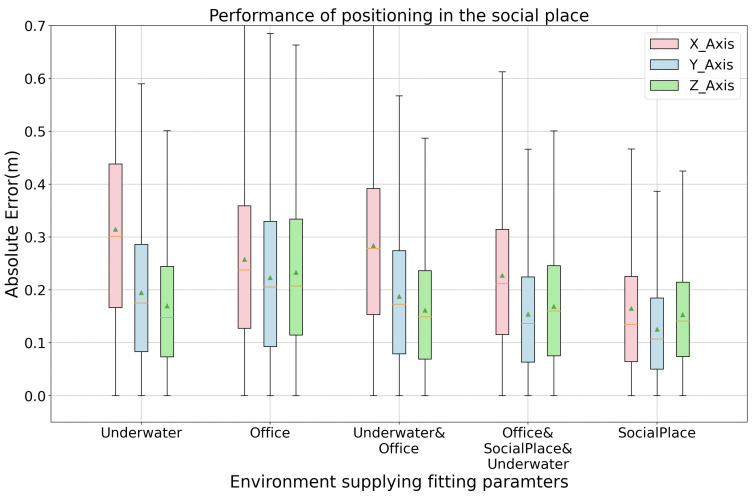
Social place positioning result with the fitting parameters from different environment.

**Figure A6:**
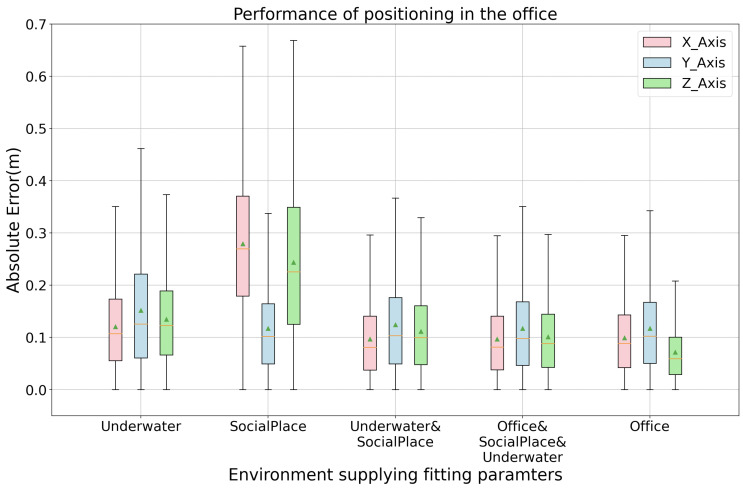
Office positioning result with the fitting parameters from different environment.

## Data Availability

The results of the system design and the basic code of this work are available from the corresponding author on reasonable request.

## Abbreviations

The following abbreviations are used in this manuscript:

ToF	Time of Flight
RSS	Received Signal Strength
LiDAR	Light and Radar
UWB	Ultra-wideband
RF	Radio Frequency
ID	Identification
MAE	Mean Absolute Error
IMU	Inertial Measurement Unit
PWM	Pulse-Width Modulation
AUV	Autonomous underwater vehicles
SLAM	Simultaneous localization and mapping
